# Burden of Severe Fungal Infections in Burkina Faso

**DOI:** 10.3390/jof4010035

**Published:** 2018-03-11

**Authors:** Sanata Bamba, Adama Zida, Ibrahim Sangaré, Mamoudou Cissé, David W. Denning, Christophe Hennequin

**Affiliations:** 1 Service de Parasitologie—Mycologie, CHUSS, Bobo-Dioulasso, Burkina Faso; hsanata@yahoo.fr (S.B.); babaibrasangare@yahoo.fr (I.S.); cisse_m@yahoo.fr (M.C.); 2 Service de Parasitologie—Mycologie, CHU Yalgado Ouédraogo, Ouagadougou, Burkina Faso; zidaadama@live.fr; 3 National Aspergillus Centre, Wythenshawe Hospital and The University of Manchester, Manchester M13 9PL, UK; ddenning@manchester.ac.uk; 4 Leading International Fungal Education (LIFE), Cheshire SK10 9AR, UK; 5 Service de Parasitologie—Mycologie, Hôpital St Antoine, Assistance Publique-Hôpitaux de Paris, 75012 Paris, France; 6 Sorbonne Universités, UPMC Univ Paris 06, INSERM, Centre de Recherche Saint-Antoine (CRSA), 75012 Paris, France

**Keywords:** Burkina Faso, aspergillosis, cryptococcosis, candidiasis, tuberculosis, AIDS

## Abstract

Because of the limited access to more powerful diagnostic tools, there is a paucity of data regarding the burden of fungal infections in Burkina Faso. The aim of this study was to estimate the incidence and prevalence of serious fungal infections in this sub-Saharan country. We primarily used the national demographic data and performed a PubMed search to retrieve all published papers on fungal infections from Burkina Faso and its surrounding West African countries. Considering the prevalence of HIV infection (0.8% of the population) and a 3.4% incidence of cryptococcosis in hospitals, it is estimated that 459 patients per year develop cryptococcosis. For pneumocystosis, it is suggested that 1013 new cases occur every year. Taking into account the local TB frequency (population prevalence at 0.052%), we estimate the prevalence of chronic pulmonary aspergillosis at 1120 cases. Severe forms of asthma with fungal sensitization and allergic bronchopulmonary aspergillosis are estimated to affect 7429 and 5628 cases, respectively. Vulvovaginal candidiasis may affect 179,000 women, and almost 1,000,000 children may suffer from tinea capitis. Globally, we estimate that roughly 1.4 million people in Burkina Faso (7.51% of the population) suffer from a serious fungal infection. These data should be used to drive future epidemiological studies, diagnostic approaches, and therapeutic strategies.

## 1. Introduction

Burkina Faso is a landlocked country of 274,200 km^2^ located in West Africa. The country lies between latitudes 9° N and 16° N and longitudes 6° W and 3° E. Ouagadougou is the capital and largest city, and Bobo-Dioulasso, the second city of the country, is an important economic center. The hydrographic network is quite developed, with rivers connected to the Volta, Comoé, and Niger basins. The climate is of the sudano–sahelian type, characterized by a rainy season (June to September) and a dry season (the rest of the year). Temperatures may vary from 16 to 45 °C. Rainfall is about 350 mm/year in the North and more than 1000 mm in the Southwest. While the North of the country is semi-desertic, most of the environment constitutes a part of the Western Sudanian Savanna.

The population reached 19,512,000 inhabitants in 2016. The urban population is 31.5% with a mean density of 64/km^2^, with much variability by region, the central and southern parts of the country having the highest concentrations. The country is characterized by a high fertility rate at 5.71 children per woman in 2017 (the median age is thus 17.3), and 65% people are under 25. The growth rate is calculated at 3.05% and the Gross Domestic Product (GDP) was estimated at $650 per capita in 2016. Despite constant progress over the last decades, the Human Development Index, at 0.402 in 2015, is still one of the lowest worldwide. This explains in part the very important migration flows, mainly to Ivory Coast, for seasonal agricultural work. The average life expectancy was estimated at 55.9 years in 2017. The infant mortality rate remains high, at 72.2 deaths/1000 live births. The health system is structured with about 1700 health and social promotion centers, 32 standard medical centers, 45 medical centers with surgical services, 9 regional hospital centers and 4 university hospital centers. In 2014, health expenditures were 5% of the GDP. The physician density was at 0.05 per 1000 population in 2012, with a bed density of 0.4 beds/1000 population. 

As for many of the surrounding countries, TB, malaria, and AIDS have been placed among the priority health problems in Burkina Faso. The risk of infectious disease is considered particularly high. This includes food and waterborne diseases (i.e., bacterial and protozoal diarrhea, viral hepatitis, typhoid fever), vector-borne diseases (i.e., malaria, dengue, yellow fever), water-contact infections (i.e., schistosomiasis), and respiratory-acquired infections (i.e., tuberculosis, meningococcal meningitis). Malaria is the predominant endemic infection in Burkina Faso. The WHO estimated the number of malaria cases at 7,890,000 (5,720,000–10,740,000) in 2016 [1]. Since 1995, a National Tuberculosis Program has been established to organize the fight against TB on a national scale. In 2016, Burkina Faso noted 5918 tuberculosis cases, 10% of which occurred in HIV-positive patients, with a mortality rate between 9% and 10%. In contrast, Burkina Faso belongs to the set of countries with a moderate prevalence of HIV infection, estimated in 2016 at 95,000 cases (0.487% of the whole population) [2]. Thanks to the setup of a national program, about 30% of patients with a CD4 cell count <200/mm^3^ benefit from antiretroviral therapy [3].

Faced with these statistics, fungal infections only represent a secondary priority at a national level. However, as already demonstrated in neighbouring countries [4], the fungal burden of serious fungal infections may be largely underestimated. This is mainly due to the limited access to adequate and reliable diagnostic tests, but also to erroneous presumptive diagnoses in favor of other prevalent infections, as reported for histoplasmosis and tuberculosis in Cameroon [5].

The main objective of this study was thus to calculate the burden of severe fungal infections in Burkina Faso by establishing reasonable estimates of their annual incidence and prevalence.

## 2. Materials and Methods

Different approaches were used to estimate the incidence and prevalence of severe fungal infections in Burkina Faso. Initially, we performed a PubMed search using the Medical Subject Headings terms: “Human”, “Infection”, “Fungi”, “Burkina-Faso” and “West Africa”. Neither limit of date nor limit of language was applied. Whatever the type of infection, if no data from Burkina Faso were available, we used incidence rates calculated or estimated in countries of West Africa or, secondarily, in the whole African continent.

For invasive opportunistic infections, we used the prevalence of the main predisposing underlying conditions, mainly AIDS [3] and TB, and the incidence of the fungal opportunistic infections in these contexts as reported in the PubMed-retrieved papers. Regarding the Invasive Fungal Infections (IFI) associated with hospital care (notably candidiasis and aspergillosis), we only considered hematology malignancies, as no information was available for solid tumors or ICU and surgical patients. No transplantation procedures are currently done in Burkina Faso.

For superficial mycoses and the immuno–allergic forms (severe asthma with fungal allergic sensitisation, allergic broncho-pulmonary aspergillosis), the gross indicators were obtained from large multinational studies or experts’ opinions [6,7,8].

Demographic data (i.e., of women, age pyramids), were obtained from the ‘Institut National de la Statistique et de la Démographie’ (INSD) [9].

For chronic infections, mainly Chronic Pulmonary Aspergillosis (CPA) and tinea capitis, we used the annual incidence to calculate a five-year prevalence.

## 3. Results

Table 1 shows the total burden of fungal infections and the number of infections classified according to the main risk factors as well as the rate per 100,000 inhabitants.

Respiratory diseases are among the most significant health problems in Burkina Faso. The sequelae of TB, including pulmonary cavities and severe forms of asthma associated with sensitization to fungal allergens, explain the high frequency of CPA, severe asthma with allergic sensitization, and Allergic Bronchopulmonary Aspergillosis (ABPA). Indeed, it is postulated that TB is the factor predisposing to the emergence of CPA in 80% of cases [10]. CPA thus appears to be the main pulmonary fungal infection in Burkina Faso. Taking into account the TB notifications (5918 cases in 2016), the estimation (9400 cases) of TB, and the frequency of pulmonary cavities post-TB, one can estimate that 284 new cases of CPA occur every year, corresponding to a five-year prevalence of 1120 patients (28.2/100,000), assuming a 15% annual mortality rate. Asthma is also one of the most common respiratory diseases worldwide. The prevalence of clinical asthma in adults in Burkina Faso has been estimated at 2.26% [11], corresponding to 225,000 patients. Some of the affected patients may develop sensitization to fungal allergens or ABPA, in the case of previous fungal colonisation. The proportion of adult asthmatics with ABPA in South Africa was 2.5% among those referred to a specialist [12], so we anticipate that 5628 people are affected in Burkina Faso. Finally, among the 10% of poorly controlled adult asthmatics, we expect that 33.5% or more are sensitized to fungi [7], and so Severe Asthma with Fungal Sensitization (SAFS) could be diagnosed in 7249 patients (41/100,000) and could be responsive to antifungal therapy.

HIV infection is the leading cause of the emergence of invasive fungal infections in Africa. Recent data from Burkina Faso suggest that cryptococcosis may complicate the course of HIV infection in 3.4% of the cases [13], thus anticipating the occurrence of 459 cases of cryptococcosis per year. Up to now, there are no incidence data available for pneumocystosis in West Africa. In a paper from Ivory Coast, 270 HIV-positive patients were included in a placebo control group (not treated), and no case of pneumocystosis was found [14]. In contrast, several papers from different African countries found an incidence of pneumocystosis (PCP) in HIV-positive patients in 1.25–49% of cases [15], and cases of pneumocystosis have been described in Burkinabe patients [16]. We used a 7.5% rate so that an annual incidence of 1013 cases could be estimated (based on a 15% rate among newly presenting AIDS patients) and so that this presentation could be spread over two years for those with CD4 counts <200 × 10^6^/mL [15,17]. In addition to these invasive infections, HIV infection is well-known as a major predisposing factor for oral thrush and its extension to the oesophagus, which may occur in 24,300 and 7450 cases, respectively. While *Histoplasma capsulatum* seems to be endemic to the region, there are no data on disseminated histoplasmosis in AIDS from Burkina Faso [18,19,20,21]. Deaths from AIDS are estimated at 3600, and it is likely that the above estimates for fungal diseases are underestimated.

Five intensive care units gathering a total of 60 beds are available in all of Burkina Faso, and there is currently no registry for cancer (hematology or solid tumors), so the incidence of hospital care-associated fungal infections is even more difficult to appreciate. We used a WHO estimation for solid tumors [22] and a previous report for leukemia from South Africa [23]. For candidemia, it is expected that this incidence would be at 3.5% for cancer (and other complex hospitalized) patients and at 1.5% for ICU/Surgical patients [24]. For invasive aspergillosis, a 10% incidence rate was considered for leukemic patients. Using these values, 906 cases of candidemia and 54 cases of Invasive Aspergillosis (IA) are expected to occur annually. It is not straightforward to estimate the number of IA cases complicating Chronic Obstructive Pulmonary Disease (COPD) admissions to hospitals, although some cases are likely.

Fungi are well-known as skin and mucosal pathogens. This is particularly the case of *Candida* as etiologic agent of vulvo-vaginitis and its recurrent form. While no data are available from Burkinabe women, it is usually considered that 6 to 9% of adult women will develop Recurrent Vulvo-Vaginal Candidiasis (RVVC) [8]. Considering the actual female population size, a simple calculation led us to estimate the occurrence of 179,002 cases of recurrent *Candida* vaginitis. Finally, it has been shown in Ivory Coast, a bordering country of Burkina Faso, that as high as 13.9% of children may be affected by tinea capitis [25]. Thus, it is expected that 1,132,781 Burkinabe children suffer from tinea capitis.

Some cases of *Histoplasma capsulatum* var. *duboisii* histoplasmosis have been reported over decades, but the prevalence cannot be estimated [18,19,20,21]. A few cases of mycetoma and rhino-facial entomophtoromycosis have also been reported from Burkina Faso [26,27,28], but to the best of our knowledge, there are no data on other subcutaneous fungal infections (i.e., chromoblastomycosis, sporotrichosis) from Burkina Faso, although they are expected.

## 4. Discussion

Fungal infections have emerged during the past decades as a major public health problem in western countries [34]. This is mainly due to new modes of patient care, such as broad-spectrum antibiotic therapy, indwelling catheters, iatrogenic immunosuppression (corticosteroid, cytotoxic chemotherapy, and, more recently, biotherapies), which are among the main predisposing factors of invasive fungal infections, such as invasive candidiasis, invasive aspergillosis, or mucormycoses. This development has been associated with significant progress in the field of diagnosis and treatment over the years, which unfortunately has not been shared with many developing countries. This hampers physicians, who therefore cannot offer therapeutic management at its best, and also explains the scarcity of local epidemiologic studies focusing on fungal infections in countries with major economic constraints, such as Burkina Faso. Indeed, confirmatory diagnosis for most Invasive Fungal Infections (IFIs) requires high-technology methods, which are usually not affordable. Currently, however, solid organ transplantation, bone marrow transplantation, and the more advanced cancer treatment programs are not yet available in Burkina Faso, probably minimizing opportunistic invasive fungal infections. At this time, there is no cancer registry in Burkina Faso, but the incidence of acute myeloblastic leukemia in adults, for example, may rise in the near future, as a consequence of cigarette smoking, occupational and environmental exposures to benzene and other pollutants, and the prescription of alkylating agents to young people with malignant diseases [35]. Lung cancer, the most frequent clinical form, is thought to only affect 166 people, but it is likely to rise with the current boom in population, the increase in smokers, and more generally, the rise in average age [22].

HIV infection represents one of the most important threats in Sub-Saharan Africa. Fortunately, Burkina Faso remains relatively preserved from the epidemic. Thus, the number of cases of pneumocystosis, whose incidence in African HIV patients is still largely unknown, and cryptococcosis remains moderate (below 500 cases). Oropharyngeal candidiasis, which is probably the most frequent opportunist infection during an HIV infection, and its extension to the oesophagus, are much more common: 24,300 and 7450 estimated cases, respectively. While several cases of histoplasmosis due to *H. capsulatum* var. *duboisii* have been described in Burkinabe patients [18,19,20,21], no case of histoplasmosis due to *H. capsulatum* var. *capsulatum* has yet been reported in Burkina Faso. However, it is important to note that this infection has been reported in several of the surrounding countries [36,37] and probably exists in Burkina Faso. Recently, Mandengue et al. suggested that it is likely that a number of cases remain undiagnosed because the clinical presentation may somewhat mimic TB [5]. It is also possible that some patients may die before the diagnosis of histoplasmosis has been mentioned. Indeed, in their study, these authors found evidence of histoplasmosis in 13% of HIV-positive patients with persistent fever and coughing associated with cutaneous lesions [5].

Respiratory diseases appear as a predominant predisposing factor for fungal complications in Burkina Faso. TB is highly endemic to Burkina Faso, as is the case for most of the West African countries. Sequelae, including cavities, favour the growth of environmental pathogens, such as *Aspergillus*. Although not currently diagnosed in Burkina Faso, cases of CPA following TB probably constitute 896 of cases, on the basis of what reported for other countries in the region, such as Senegal [4,10]. In our study, the number of CPA cases may be underestimated, as other lung diseases, such as emphysema, pneumothorax, sarcoidosis, asthma, and others, predispose to CPA. Moreover, as radiologic symptoms are not specific, and *Aspergillus* may be a culture contaminant, *Aspergillus* infections should be confirmed using a specific serology, which unfortunately is not available in Burkina Faso. Disturbingly, the five-year mortality rate of CPA is reported as 50–85% [10,38]. Asthma is also present worldwide, and patients with the most severe forms are more prone to be colonized and allergic to airborne fungi. This can lead to fungal sensitization or more rarely to authentic ABPA. The impact of IgE-mediated sensitization as a cause or consequence of asthma is uncertain [39], but it is associated with more severe attacks and possibly asthma deaths [12]. In contrast, it is well known that ABPA, which manifests as acute exacerbations, may also lead to pulmonary fibrosis and chronic respiratory failure [39]. Again, absolutely no information regarding these long-term complications are available in Burkina Faso.

Muco-cutaneous fungal infections are also very common, often without specific underlying medical conditions. A recent study reports a 22.71% prevalence rate of candidal vulvovaginitis in Burkinabe pregnant women [40], and *C. albicans* was demonstrated in 48.76% of Burkinabe women with abnormal vaginal discharge [41]. The recurrent form occurs worldwide and results in a huge number of infections, both in developed and developing countries, such as Burkina Faso [4,42]. Taking into account the local healthcare facilities, it is unlikely that women have access to dedicated medical advice for such kinds of infection. Finally, tinea capitis is very common in children of sub-Saharan countries. In a large study conducted in Ivory Coast, 13.9% of 17,745 children were found to be positive, mainly with anthropophilic dermatophytes, such as *Trichophyton soudanense* and *Microsporum langeroni* [25]. It is highly likely that a similar pattern and frequency would be seen in Burkina Faso.

## 5. Conclusions

In conclusion, with almost 1.4 million cases within the 19.5 million people, severe fungal infections affect 7.51% of the population of Burkina Faso. This result is overall comparable to that of Senegal, another West African country [4]. It is important to note that only very few local studies are available to support this estimation. The reasons for this hiatus are directly related to the limited access to both more powerful diagnostic tools required for some of these infections and more basic diagnostic tools, such as microscopy and fungal culture. These conditions lead to a relative lack of knowledge on fungal infections from medical staff. This study should help to drive some of the future studies needed to implement diagnostic algorithms and finally to improve the quality of life of the Burkinabe population.

## Figures and Tables

**Table 1:** Estimate of the burden of fungal diseases in Burkina Faso.

Underlying Condition	Assumed Incidences Used for the Calculation of Fungal Burden with References	Fungal Infections	Annual Incidence	Rate/100 K
**Respiratory diseases**	CPA: assumes that TB is the underlying cause of CPA in 80% TB: prevalence: 5800 (notified)–9400 (estimated); 0.85 with pulmonary involvement; 0.93 with pulmonary cavity; 15% mortality rate	CPA	1120 ^1^	28.2
	CPA: incidence in cavitary TB: 0.12–0.22; incidence in non-cavitary TB: 0.01–0.04 SAFS: Asthma: 0.0226 of adult population [6]	SAFS	7429	41
	1/3 of the 10% worst asthmatic patients develop SAFS ABPA: 0.025 of adult asthmatic pts [7]	ABPA	5628	31.1
**AIDS**	95,000 HIV+/AIDS pts [29] 57% not receiving ARV with CD4 cell count <350/mm^3^ Oral candidiasis: 90% of HIV pts with <200 CD4 [30]	OPC	24,300	134
	Oesophageal candidiasis: 20% of pts not on ARVs and CD4 <200, and 0.5% of those on ARVs [31]	Oesophageal candidiasis	7450	41.1
	Cryptococcosis: incidence 0.034 [13]	Cryptococcosis	459	2.5
	Pneumocystosis: incidence 0.095 [32]	Pneumocystosis	1013	5.6
**Care-associated infections**	Invasive aspergillosis: 10% of haematological malignancies	Invasive aspergillosis	54	0.3
	AL mean incidence: 0.0135 [23]	Candidemia	906	5.0
	Invasive candidiasis: Candidemia: 3.5% of cancer pts, 1.5% of surgical/ICU pts [24] + Peritonitis: 0.5*candidemia cases of surgical/ICU pts [33]	*Candida* peritonitis	136	0.75
**Mucosal, skin, and superficial fungal infections**	RVVC: roughly 6% of adult women [8]	RVVC	179,002	1977
	*Tinea capitis*: assumes a 13.9% prevalence in children under 16 [25]	Tinea capitis	1,132,781	6255
**Total**			1,360,280	

^1^ Prevalence. pts: patients. AL: Acute Leukemia. CPA: Chronic Pulmonary Aspergillosis. OPC: Oro-Pharyngeal Candidiasis. SAFS: Severe Asthma with Fungal Sensitization. RVVC: Recurrent Vulvo-Vaginal Candidiasis; * mean time.
